# Rifaximin is an effective alternative to metronidazole for the treatment of chronic enteropathy in dogs: a randomised trial

**DOI:** 10.1186/s12917-016-0851-0

**Published:** 2016-10-06

**Authors:** Alessandro Menozzi, Manuel Dall’Aglio, Fausto Quintavalla, Luca Dallavalle, Valentina Meucci, Simone Bertini

**Affiliations:** 1 Department of Veterinary Science, University of Parma, Strada Del Taglio 10, 43126 Parma, Italy; 2 Veterinary Practitioner, Parma, Italy; 3 ATI Pets Srl, Fatro Group SpA, Ozzano dell’Emilia, Bologna, Italy; 4 Department of Veterinary Science, University of Pisa, Pisa, Italy

**Keywords:** Dog, Chronic enteropathy, Rifaximin, Metronidazole, CIBDAI

## Abstract

**Background:**

A clinical trial was conducted in order to assess the efficacy of rifaximin, a broad-spectrum antibiotic with negligible gastrointestinal absorption, in comparison with metronidazole, a commonly employed antimicrobial drug, in dogs with chronic enteropathy. Twenty-four pet dogs were randomly enrolled into two different groups: MET group (10 dogs) and RIF group (14 dogs). Dogs of MET group received metronidazole 15 mg/kg q12h for 21 days by oral route, whereas dogs of RIF group, were given rifaximin 25 mg/kg q12h for 21 days by oral route. Clinical signs of disease were evaluated the day before the beginning of drug administration (D0), and at the end of treatment (D21), by means of Canine IBD Activity Index (CIBDAI). Blood levels of C-reactive protein (CRP) at D0 and D21 were also measured, as another parameter of treatment efficacy. The primary outcome measure of efficacy was the complete remission at D21, defined as a 75 % or greater decrease of CIBDAI; secondary outcome measures were the variation of mean CIBDAI scores, of mean CRP serum levels, and any observed adverse effect from D0 to D21.

**Results:**

Treatment with metronidazole or rifaximin greatly improved the clinical signs of disease in each group: in MET group the complete remission was achieved in 8 of 10 dogs (80.0 %), and partial remission in 2 subjects (20.0 %). In RIF group, 12 of 14 dogs showed complete remission (85.7 %), and the remaining 2 dogs were in partial remission (14.3 %). There were also significant decreases of CIBDAI scores (*P* = 0.002 and *P* = 0.0002 for MET and RIF, respectively), and CRP levels (*P* = 0.002 and *P* = 0.0001 for MET and RIF, respectively) compared to pre-treatment values in both groups. No significant difference, however, was found when comparing MET and RIF groups. No relevant side-effect was reported during the trial with either drugs.

**Conclusions:**

The present study showed, for the first time, that oral rifaximin could represent an effective alternative to metronidazole for the induction of clinical remission in dogs with chronic enteropathy.

**Electronic supplementary material:**

The online version of this article (doi:10.1186/s12917-016-0851-0) contains supplementary material, which is available to authorized users.

## Background

Inflammatory bowel disease (IBD) is a generic name which includes different chronic inflammatory disorders affecting the gastrointestinal tract in human patients, the most important of which are Crohn’s disease (CD) and ulcerative colitis (UC). Dogs and cats are also susceptible of developing chronic gastro-intestinal inflammation which share several aspects with human IBD [1]. In these species the most frequent histological forms of chronic enteropathy are lymphocytic-plasmacytic enteritis (LPE) and eosinophilic gastroenteritis (EGE), and while the clinical and histological features of IBD in small animals are not always closely akin to those of CD and UC, the pathogenesis of such diseases is thought to be very similar. Indeed, it seems that an abnormal response of the immune system due to a loss of tolerance to different luminal antigens is the key pathogenetic event of both human and animal IBD [2–4]. Though the pathogenesis is still not fully understood, it is generally accepted that IBD has a multifactorial etiology, and that genetic predisposition, together with environmental factors and a derangement of gut epithelial barrier, are all contributing to the generation and/or perpetuation of inflammation. Several studies suggest that a hyper-reactivity of the immune system in the gut against normally well-tolerated antigens, like food components or bacterial microbiota, ignites the inflammatory response which is then responsible for gastrointestinal damage [2, 3]. Moreover, the inflammation is carried on and exacerbated by various problems such as resistance to apoptosis of lymphocytes and recurrent exposure to luminal antigens due to an enhanced mucosal permeability [5–7].

The treatment of CD or UC in human patients, as well as in animals affected by IBD, is mainly based on the use of anti-inflammatory and immunosuppressant drugs (corticosteroids, 5-aminosalicylates, azathioprine, cyclophosphamide) in order to normalize the up-regulated immune response. Even though the drugs prescribed to dogs and cats affected by IBD are often the same employed in human patients, some forms of chronic enteritis in these species respond to simple diet modification or to probiotics and antimicrobial drugs [8, 9]. There are several evidences of the importance of bacterial microbiota in the pathogenesis or worsening of IBD [10–12] but, whereas antibiotics seem to ameliorate experimental bowel inflammation [13–15], the results obtained against CD or UC in clinical trials with antibacterial agents are not very encouraging, except for some positive effects obtained with metronidazole and ciprofloxacin [16, 17]. Metronidazole has proven to be effective also in small animal IBD [18], suggesting that bacteria may play a role in chronic gastrointestinal inflammation of dogs and cats, even if the real weight of antimicrobial action compared with the immunomodulating activity possessed by this drug has to be clarified yet.

Rifaximin (4-deoxy-4′-methylpyrido[1′,2′-1,2]imidazo-[5,4-c]-rifamycin SV) is a semisynthetic rifamycin endowed with a wide spectrum of antibacterial activity, and it is virtually non-absorbable by oral route [19], thus granting high efficacy and low incidence of side-effects [20]. Previous studies showed that rifaximin was more effective than other antibiotics in human patients with irritable bowel syndrome (IBS), and induced remission of moderately active CD in 2/3 of the subjects treated [21, 22]. Rifaximin was also more active than metronidazole in human patients with intestinal bacterial overgrowth [23]. Even though an efficacy of this drug in human IBD and in experimental models of intestinal inflammation [15, 24, 25] was demonstrated, as for metronidazole, the reasons underlying this beneficial activity are still unclear.

The aim of the present study was to investigate the therapeutic effects of oral rifaximin in dogs affected by chronic entheropathy, compared with those of metronidazole, an antimicrobial and antiprotozoal drug commonly employed in the therapy of chronic intestinal inflammation in small animals.

## Methods

### Animals

**Fig. 1:**
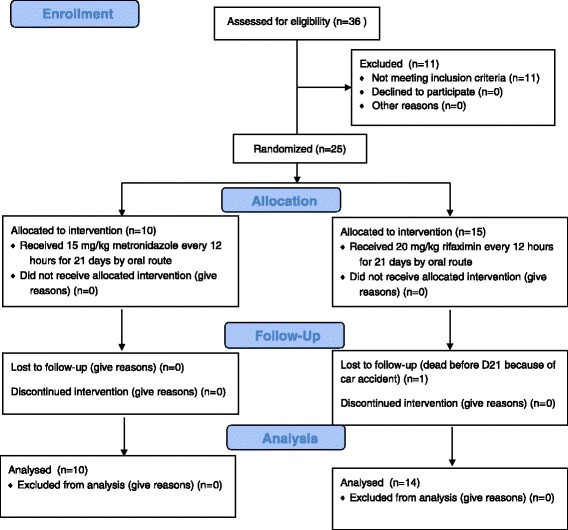
Flow chart of trial enrolment and treatment protocol

Thirty-six pet dogs, males and females, diagnosed with chronic enteropathy both by clinical and histological evaluation, were assessed for eligibility, and 25 of them, were enrolled in the clinical trial (Fig. 1). The sample size was determined on the basis of a previous similar study [26]. The study was conducted between September 2014 and March 2016, at the Veterinary Hospital of the University of Parma, apart from the histologic and biochemical analysis, which were performed at the Idexx Laboratories. The animals were included in the trial on the basis of the following criteria: clinical signs of gastrointestinal disease lasting for more than 3 weeks; lack of a relevant improvement after at least 4 weeks of a commercial elimination diet (with the recommendation to the owner to feed the dogs exclusively with the prescribed diet), or after the treatment with anthelmintic drugs, antibiotics (amoxicillin/clavulanic acid or enrofloxacin), spasmolytic and antidiarrhoeal agents; failure to detect other causes of disease after a thorough diagnostic protocol. The diagnostic evaluation for all dogs included at least CBC, serum biochemistry and urinalysis, three daily consecutive faecal examinations for endoparasites (both by direct smear and by flotation technique), faecal chymotrypsin and serum trypsin-like immunoreactivity, abdominal ultrasonography. Exclusion criteria were a previous treatment with immunosuppressant drugs or the presence of hypoalbuminemia (9 dogs); moreover, 2 dogs with a suspected diagnosis of primary lymphangiectasis were also not included in the study. The diagnosis of chronic enteropathy was confirmed in all subjects by means of endoscopy (Fujinon EG-250WR5), and subsequent histologic evaluation of multiple biopsies.

### Trial design

The dogs were randomly assigned to two different groups, named MET (metronidazole) and RIF (rifaximin), with an allocation ratio of 1:1. MET group was composed by 10 dogs, while the dogs assigned to RIF group were 15. One of the dogs belonging to RIF group died before the conclusion of the treatment period, and was therefore excluded from the results. Each dog meeting the criteria for enrolment in the trial was assigned to MET group if its name was composed by an even number of letters, or to RIF group if the name was instead made of an odd number of letters.

Metronidazole was administered by oral route to the dogs of MET group, at the dose of 15 mg/kg q12h for 21 days, whereas rifaximin, at the dose of 25 mg/kg q12h for 21 days, was given by oral route to the dogs enrolled in RIF group. Metronidazole was administered at a dose in the range commonly employed in the therapy of IBD in dogs (10–25 mg/kg q12h) [18], whereas the dosage used for rifaximin was chosen on the basis of a previous work [15]. The dogs were examined for the assessment of the severity score of the disease by a member of our research group, blind to the treatment, the day before the beginning of drug administration (D0), at day 15, and at day 21 (D21), at the end of metronidazole or rifaximin administration period. All dogs received also ranitidine (2 mg/kg q12h), and metoclopramide (0.3 mg/kg q12h until day 15). A supplement of vitamin B_12_ (cyanocobalamin 250–1000 μg i.m. according to the weight) was administered once a week to the dogs. In order to exclude any influence of food on the evaluation of drug efficacy, all dogs were fed with a home-made diet which consisted exclusively of boiled chicken meat and rice.

Blood samples were collected from each dog at D0 (pre-treatment level) and at D21 (post-treatment level) for the measurement of C-reactive protein (CRP), which is an acute-phase protein produced by the liver in response to inflammation, infection and tissue injury, and whose decrease from pre-treatment serum level is considered a suitable marker of the improvement of the intestinal inflammatory status [27].

### Drugs

A commercially available oral formulation of metronidazole, Flagyl® 250 mg tablets (Zambon Italia Srl, Milano, Italy) was employed in the study. Rifaximin 250 mg divisible tablets were kindly supplied by Ati Pets Srl, Fatro Group SpA, Ozzano dell’Emilia, Bologna, Italy.

### Clinical evaluation and measures of outcome

The severity of the disease was scored by means of Canine IBD Activity Index (CIBDAI), which is a widely accepted index of mucosal inflammation in canine IBD [28]. Briefly, six salient clinical signs (attitude/activity, appetite, vomiting, stool consistency, stool frequency, weight loss) were assessed and scored 0 through 3, according to the degree of alteration from normal in each patient. The obtained scores were then summed in order to obtained a cumulative CIBDAI score, indicating the severity of the disease: clinically insignificant (0–3), mild (4–5), moderate (6–8) or severe (9 or greater).

The primary outcome assumed as therapeutic response to the treatment was clinical remission at D21, according to a previous study [27]. Complete remission, was defined as 75 % or greater decrease of CIBDAI score with respect to pre-treatment (D0), while a reduction of CIBDAI <75 % but >25 % was considered as a partial remission. Secondary measures were the percent variation of mean CIBDAI score from D0 to D21 in both groups, and the modification of pre-treatment mean CRP serum concentration at the end of treatment. Any adverse effect observed during the trial was also considered as a secondary outcome.

### Histology

**Fig. 2:**
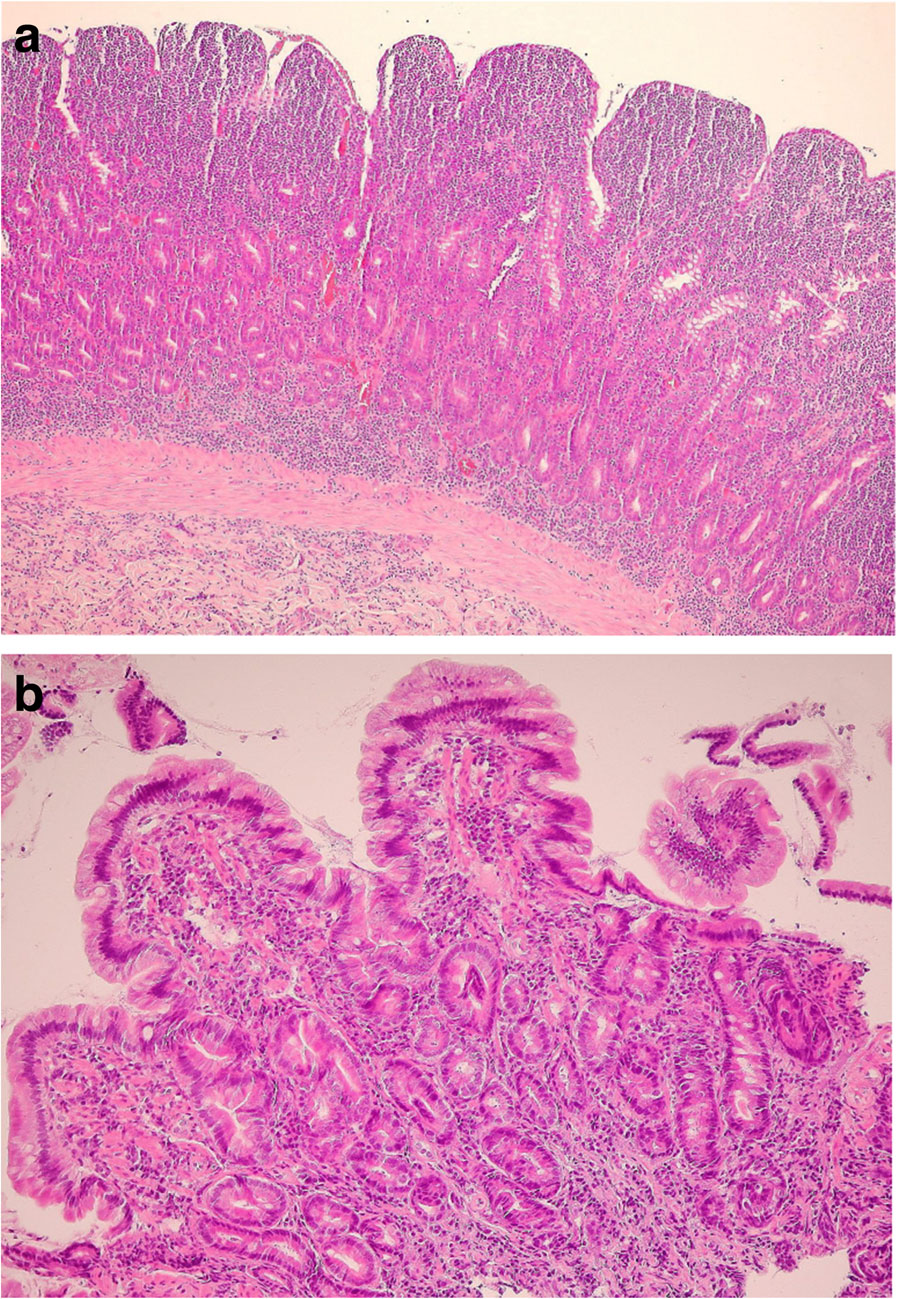
Histologic features of small intestine sections of dogs with LPE. Panel **a**: Duodenal mucosa of a dog enrolled in MET group (CIBDAI score 11 at D0) with severe lymphoplasmacytic inflammation. The infiltrate of inflammatory cells is extending from the tip of the villi, through the lamina propria, to the muscularis mucosae (haematoxylin and eosin; 20×). Panel **b**: Duodenal mucosa of a dog enrolled in RIF group (CIBDAI score 3 at D0), with moderate lymphoplasmacytic inflammation. The villi are blunted, and the inflammatory cells are scattered into the lamina propria (haematoxylin and eosin; 40×)

Tissue samples collected during endoscopy, after fixation in 10 % buffered formalin, were processed according to an automatised method (Tissue-Tek Xpress 120, Sakura Finetek Europe B.V., Flemingweg, the Netherlands). Serial paraffin sections (2 μm) were then prepared, and stained with haematoxylin and eosin for morphological examination. Histologic evaluation was performed by two observers, blind to the treatment (Fig. 2).

### C-reactive protein assay

CRP was measured by means of an immunoturbidimetric assay (Randox Canine CRP, Randox Laboratories Ltd., U.K.). The reference range for CRP level in healthy dogs measured with this assay is 0–10.7 mg/l.

### Statistical analysis

The two different treatment groups were compared about two outcome results (CIBDAI and CRP serum level). Comparisons within the same group between pre- and post-treatment values were made by means of non-parametric Wilcoxon matched pairs signed rank tests; whereas when the differences between MET group and RIF group were analysed, cumulative distributions were compared with Kolmogorov-Smirnov unpaired tests. All statistical measures were performed using a commercial software (GraphPad Prism, ver. 6.05, GraphPad Software Inc., La Jolla, CA, U.S.A.).

## Results

MET group consisted of 10 dogs (3 cross-breeds, 2 German Shepherds, 1 Rottweiler, 1 Boxer, 1 Golden Retriever, 1 Pinscher, 1 Épagneul Breton), whereas RIF group was composed by 14 dogs (5 cross-breeds, 2 German Shepherds, 1 Irish Setter, 1 Poodle, 1 Dachshund, 1 Boxer, 1 Dogo Argentino, 1 Maltese, 1 Bichon Frisè). Mean weight at D0 was 21.65 ± 11.61 kg (range: 6–45) for dogs of MET group, and 16.32 ± 8.47 kg (range: 5–28) for dogs belonging to RIF group.

Histologic features of biopsies of all dogs showed an inflammation with lymphocytic-plasmacytic infiltrate of small intestinal mucosa, ranging from mild to severe, often with alterations of the structure of villi (blunted or fused). In particular, in MET group the number of dogs with mild, moderate or severe inflammation, were 1, 6, and 3, respectively. In the same group 5 dogs presented blunted, and one dog fused villi. In RIF group, 4 dogs showed mild, 7 moderate, and 4 severe inflammation. In this group, 8 dogs showed blunted, and 2 fused villi.

**Fig. 3:**
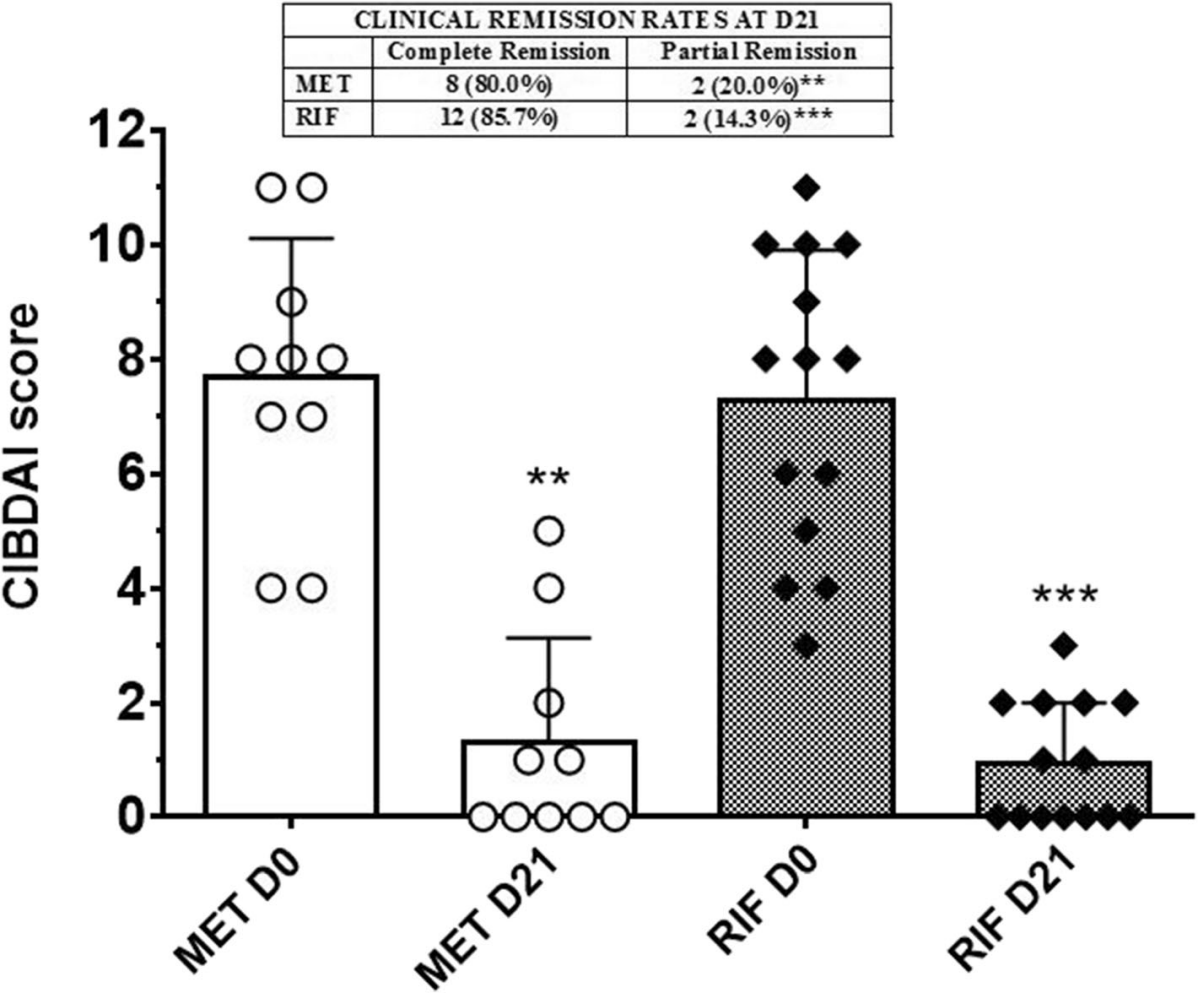
Effect of metronidazole and rifaximin administration on clinical remission rates (*box*) and CIBDAI scores (*columns* and *symbols*) of dogs in both treatment groups. *Columns* in the graph represent mean ± SD, while *symbols* are individual CIBDAI scores of dogs in MET group and RIF group at D0 and at D21. **P < 0.001 D21 vs D0; ***P < 0.0001 D21 vs D0

At D21 in dogs of MET group the complete remission was achieved in 8 of 10 animals (80.0 %), while 2 subjects were in partial remission (20.0 %). In RIF group, 12 of 14 dogs showed complete remission (85.7 %), and the remaining 2 dogs were in partial remission (14.3 %) (Fig. 3).

Mean CIBDAI scores at D0 were 7.70 ± 2.41 (range: 4–11) and 7.29 ± 2.61 (range: 3–11) for MET and RIF group, respectively (*P* = 0.921). Treatment with metronidazole or rifaximin, along with diet change, greatly improved the clinical signs of disease in each group at D21, as shown by the significant decrease of CIBDAI values, compared to pre-treatment values (1.30 ± 1.83, *P* = 0.002, and 0.92 ± 1.11, *P* = 0.0002, respectively) (Fig. 3). There was, however, no significant difference between MET and RIF groups in the percent decrease of CIBDAI scores from pre-treatment level to D21 (85.84 vs 88.34 %, respectively, *P* = 0.999).

**Fig. 4:**
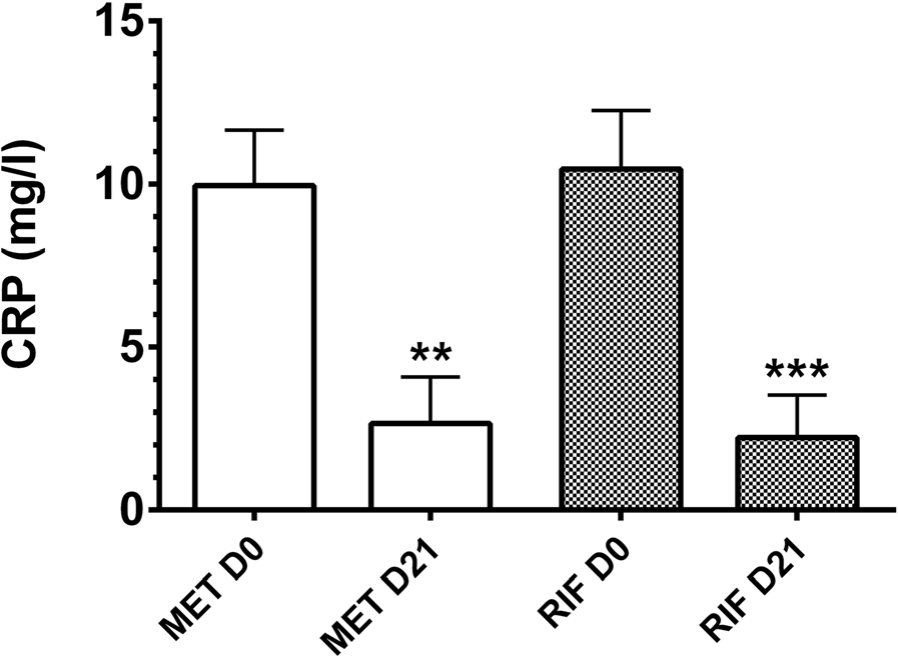
Effect of metronidazole and rifaximin administration on CRP serum levels of dogs in MET group and RIF group. *Columns* in the graph represent mean ± SD. **P < 0.001 D21 vs D0; ***P < 0.0001 D21 vs D0

Mean CRP levels at D0 of dogs in MET group and RIF group were 9.95 ± 1.71 mg/l and 10.46 ± 1.80 mg/l, respectively (*P* = 0.974). After 3 weeks of both metronidazole and rifaximin administration (D21), mean CRP serum levels were significantly reduced (2.66 ± 1.42 mg/l, *P* = 0.002, and 2.23 ± 1.30 mg/l, *P* = 0.0001, respectively) (Fig. 4). The decrease, expressed in percentage, of mean CRP concentration in MET group was 74.07 ± 12.45 %, whereas it was 79.75 ± 10.00 %, in dogs of RIF group, without achieving, anyway, a significant difference (*P* = 0.446).

No significant adverse effect was observed during the treatment period in both groups of dogs. Raw data of CIBDAI scores, remission rates, dog weights, and CRP serum levels are reported in Additional file 1.﻿

## Discussion

Treatment of canine chronic enteropathies is often including antibacterial agents, based on the evidences of an efficacy of antibiotic therapy in ameliorating clinical signs of disease, and of a possible pathogenetic role of bacterial antigens in the generation and perpetuation of inflammatory status in the gastrointestinal mucosa [29, 30]. The present study was aimed to compare the clinical efficacy of rifaximin, a non-absorbable rifamycin, with respect to metronidazole, an antibiotic commonly prescribed against canine IBD, in dogs in which non-hypoproteinemic chronic enteropathy was diagnosed. After the administration of either antibiotic for 21 days, and diet modification, clinical signs were greatly improved, as shown by the number of dogs which achieved complete remission, and by the significant fall of mean CIBDAI scores in both groups. There was, however, no significant difference in efficacy between metronidazole and rifaximin, which demonstrated to be equally effective in this clinical trial.

The beneficial effect of metronidazole and rifaximin against intestinal inflammation was confirmed by the significant decrease of mean CRP serum concentration at D21 with respect to pre-treatment level in both groups, as the correlation of this acute-phase protein with inflammatory status in human and dog IBD has been observed in several studies [27, 31]. However, since CRP is a non-specific marker of inflammation, the interpretation of this result should be prudently considered without further studies.

To date, little is known about the species of microbiota which may play a role in the pathogenesis of canine IBD. Whereas many studies have shown that an alteration of gut microbiota takes place in dogs and cats with chronic intestinal inflammation, and that this dysbiosis is similar to those observed in human IBD or in animal models of intestinal inflammation, the composition of such abnormal bacteria population seems to be variable, thus making the choice of a selective antibiotic treatment a very difficult task [32]. In the present study, rifaximin, a broad spectrum antibiotic, was effective as metronidazole, whose activity is directed only against anaerobes. This result seems to suggest that anaerobic bacteria could be particularly important in the pathogenesis of chronic diarrhoea in dogs; as a matter of fact, a recent study [33] evidenced an increased number of anaerobic species in the mucosa of dogs with chronic enteropathies. By contrast, other studies conducted in dogs with IBD, found either a reduction of anaerobic bacteria [32] or no significant alterations compared to healthy subjects [26]. Interestingly, it was observed that rifaximin was able to enhance the growth of beneficial bacteria such as *Bifidobacteria* or *Faecalibacterium praunsnitzii* [34], and that a reduced population of *Faecalibacterium* in particular, which seems to exert a protective activity against intestinal inflammation [35], is usually present in human patients and dogs with IBD [26, 36, 37]. However, metronidazole, which resulted effective as rifaximin in this clinical trial, is by contrast known to decrease anaerobes such as *Faecalibacterium*, and thus the protective role of some intestinal microbiota against inflammation remains to be clarified.

Metronidazole, a nitroimidazole antibiotic, is widely used for the treatment of IBD in dogs, both alone or in combination with corticosteroids or immunosuppressant drugs, even though its efficacy was investigated only in few published studies [26, 27, 38, 39], and the mechanisms by which it improves the clinical signs of the disease are still to be fully understood. Likewise, although abundant experimental evidence supports the hypothesis that bacteria participate to the pathogenesis of IBD in humans, and metronidazole was shown to be effective in reducing disease severity in patients with CD [16, 40], and equal or even superior to sulphasalazine in another study [41], the real utility of this drug, or the reasons underlying its beneficial activity, remain controversial. It has been proposed that metronidazole could be effective for its immuno-modulating properties rather than for a simple antibacterial activity, since this drug is able to suppress cell-mediated immunity [42, 43].

As for rifaximin, an open-label study on IBD patients demonstrated an efficacy in decreasing disease activity index [44], and a significant advantage of a 12-week treatment with this antibiotic over placebo in inducing clinical remission in mild-to-moderate CD [22]. Furthermore, rifaximin was proved to be effective in preventing bacterial translocation into mesenteric lymph nodes, and to ameliorate experimental colitis in mice [15].

Recently, a protective role by nuclear receptors like pregnane X receptor (PXR), peroxisome proliferator-activated receptor-γ and liver X receptor in IBD was suggested by some studies [45–47]. In particular, PXR ligand pregnenolone-16α-carbonitrile was able to ameliorate dextran sodium sulfate (DSS)-induced colitis in mice, and such effect was related to a reduced expression of NF-kB transcription factor [48]. Through NF-kB inhibition, a repression of its target genes is obtained, leading to a decrease in the production of pro-inflammatory mediators like TNF-α, IL-1β and IL-6. Rifampicin and rifaximin are known agonists at PXR [49], and the latter has been recently investigated for its effects on experimental colitis in the mouse, showing to protect against DSS-induced damage by inhibiting NF-kB signaling inflammatory cascade [24]. Since NF-kB activation has been observed in dogs affected by LPE [50, 51], it is possible that rifaximin efficacy could be due, at least in part, to its anti-inflammatory activity. It is interesting to note that, tylosin, a macrolide antibiotic employed in the treatment of chronic diarrhoea in dogs [38], and which was effective against experimental colitis in rats [52], is also endowed with anti-inflammatory properties [53], thus strengthening the hypothesis that antimicrobic agents which are effective against chronic intestinal inflammations in dogs, might aim at two distinct targets, the bacteria in the bowel lumen, which trigger the immune response, and the immune response itself.

In this clinical trial, no significant side-effect was observed with either antibiotic treatment during the 21-days administration. Several adverse effects are nevertheless reported in literature following metronidazole administration, and neurological toxicity is frequently reported [54, 55]. Even though data about rifaximin safety in dogs are thus far lacking, this drug was devoid of adverse effects when administered to rats up to 100 mg/kg for 6 months [56], and was shown to be safe and effective in children with IBD [57], most probably because of its negligible intestinal absorption through healthy or inflamed mucosa. Indeed, rifaximin absorption was not modified by intestinal inflammation in indomethacin-induced enteropathy in rats [58]. However, a long-term evaluation of potential adverse effects in dogs caused by rifaximin administration is necessary in order to thoroughly assess the safety of this antimicrobic drug. Negative effects due to antibiotic-induced alteration of intestinal microbiota cannot be in fact excluded, since, for instance, it was observed in a recent study that antibiotic-responsive dogs with chronic enteropathies have worse outcome compared to those treated only with diet modification [59].

A limitation of this clinical trial could be the relatively small number of dogs evaluated, which might have not allowed to unveil small differences of efficacy between the two antibiotic treatments; moreover, a single dosage for both rifaximin and metronidazole was employed, thus possibly clouding the evidence of a dose-dependent effect. Metronidazole, however, was administered at a commonly employed dose, whose efficacy and relative lack of major side-effects is generally accepted; as for rifaximin instead, possible dose-related differences in efficacy or adverse effects could not be ruled out. Another limitation of the present study is represented by the possible therapeutic role played by the home-made diet, as it is a low-fat, highly digestible one, and some of the dogs could have responded to diet modification. A future clinical trial including a group of dogs treated only with this kind of home-made diet, in comparison with antibiotic administration, would be useful to better enlighten the therapeutic effect of rifaximin. Furthermore, a contribution of antiemetic drugs to the improvement of CIBDAI scores cannot be excluded. It would also have been very important to include in the study the effects of drug treatment in a longer period of time, as chronic enteropathies are commonly relapsing after therapy is discontinued; the evaluation at the conclusion of the 3 weeks of treatment is, anyway, usually adequate to assess the response to drugs, at least to evaluate the ability to induce the remission of clinical signs [28]. Further studies with different doses of rifaximin, and over a longer period of time, will allow to better understand the efficacy and safety profile of this new therapeutic approach to chronic intestinal inflammation in dogs. Moreover, supplementary studies about rifaximin activity on different intestinal microbiota could be crucial to broaden the knowledge on the clinical potential of this antibiotic against chronic enteropathies in dogs.

## Conclusions

This study suggests, for the first time, that rifaximin may indeed represent an attractive alternative to metronidazole for the therapy of chronic intestinal inflammations in dogs.

## Additional file

Additional file 1: Raw Data Rifaximin BMC Vet Res.xlsx (Raw Data Spreadsheet). Raw data of CIBDAI scores, remission rates at day 21, dog weights, and C-reactive protein levels. (XLSX 10 kb)

## Abbreviations

CBC: Complete blood count
CD: Crohn’s disease
CIBDAI: Canine IBD activity index
CRP: C-reactive protein
DSS: Dextran sodium sulfate
EGE: Eosinophilic gastroenteritis
IBD: Inflammatory bowel disease
IBS: Irritable bowel syndrome
IL-1β: Interleukin-1β
IL-6: Interleukin-6
LPE: Lymphocytic plasmacytic enteritis
MET: Metronidazole group
NF-kB: Nuclear factor kappa-light-chain-enhancer of activated B cells
PXR: Pregnane X receptor
RIF: Rifaximin group
TNF-α: Tumour necrosis factor alpha
UC: Ulcerative colitis
